# Development and validation of a scale for assessing community adults’ knowledge, attitude and practice toward adult snoring

**DOI:** 10.3389/fpubh.2026.1754193

**Published:** 2026-03-10

**Authors:** Shaoping Yang, Siyan Guo, Xiaokai Wang, Qiufang Li, Jia Wang, Kaiwen Liu, Siyue Wang, Hao Zhang

**Affiliations:** 1 School of Nursing and Health, Zhengzhou University, Zhengzhou, China; 2 The First Affiliated Hospital of Zhejiang University School of Medicine, Hangzhou, China; 3 The First Affiliated Hospital of Zhengzhou University, Zhengzhou, China

**Keywords:** adult, community, health knowledge, attitudes, practice, instrument, snoring

## Abstract

**Introduction:**

This study aimed to develop and validate a scale for assessing community adults’ knowledge-attitude-practice toward Adult Snoring.

**Methods:**

A total of 1,198 Chinese community adults were included in the development of this scale. An initial item pool was first constructed through literature review and qualitative interviews; after item optimization through Delphi expert consultation and pre-survey, the scale structure was determined through item screening, exploratory factor analysis, and confirmatory factor analysis, with reliability and validity tests also completed.

**Results:**

The scale comprised 32 items, with 18, 7, and 7 items, respectively, for knowledge dimension, attitude dimension, and practice dimension. Exploratory and confirmatory factor analyses supported three dimensions and five factors (basic cognition, causes and risk factors, snoring harm, attitude, and practices). Internal consistency for the total scale was high (Cronbach’s *α* = 0.962). The construct reliability of the five factors was 0.911, 0.912, 0.908, 0.920, and 0.910, respectively. The intraclass correlation coefficient of test–retest reliability was 0.88, providing support for the stability of the scale.

**Conclusion:**

The scale demonstrates excellent psychometric properties, making it a reliable and valid tool for assessing community adults’ knowledge, attitude, and practice toward Adult Snoring. Identifying gaps in knowledge, attitudes, and practices about adult snoring among community adults supports the development of effective prevention-oriented health promotion strategies.

## 1 Introduction

Snoring is a highly prevalent nocturnal phenomenon in community settings, often witnessed by bed partners or family members as an audible vibratory sound during sleep (1, 2). In many cultures, including that of China, it is commonly misinterpreted as a sign of deep or restful sleep rather than a potential health concern (3). However, evidence indicates that snoring may represent the earliest community-perceivable manifestation along the continuum of sleep-disordered breathing (SDB), which encompasses a spectrum ranging from primary snoring to obstructive sleep apnea–hypopnea syndrome (OSAHS) (4, 5). Critically, snoring is a sentinel symptom that can be recognized in everyday life (2), making it a unique entry point for early public health.

However, the systemic health implications of snoring remain poorly recognized despite its high visibility in community settings (6, 7). Evidence has indicated that snoring is associated with underlying pathophysiological disturbances such as intermittent hypoxia, intrathoracic pressure swings, and sympathetic overactivity, which lead to many chronic conditions (including hypertension, type 2 diabetes, and ischemic stroke) (8–11). Yet, in real-world practice, these downstream diseases are often given sufficient attention, their upstream homologous issue, snoring, is frequently overlooked (12, 13). This reflects a fundamental gap in community residents’ awareness of snoring as a potential health risk. Consequently, there is an urgent need to assess and address these gaps using valid, community-appropriate measurement instruments.

Existing instruments usually focus on treatment adherence and symptom severity in OSAHS (14–17), thereby overlooking snoring as a common and persistent indicator of systemic risk and the priorities of community-based public health. Furthermore, they generally exclude non-snorers, who represent a critical target group for primary prevention, and overlook the potential role of family members as facilitators of health-seeking behavior. To date, no validated KAP scale has been developed to comprehensively capture community adults’ understanding of snoring as a perceivable indicator of systemic health risk.

To address this gap, this study defines “adult snoring” as an audible vibratory sound produced by the upper airway during sleep, as recognized by the individual or their close contacts in everyday life (1). This definition reflects how snoring is commonly experienced and interpreted in community settings (13). Accordingly, the construct serves as a foundation for public health action, encompassing all community adults aged 18 years or older, regardless of personal snoring status, health condition, or history of medical consultation. Critically, this study conceptualizes snoring not as a proxy for OSAHS, but as a population-level risk signal that warrants early action across cognitive, attitudinal, and behavioral domains.

This study developed and validated a knowledge, attitudes, and practices (KAP) scale to assess community-dwelling adults’ perceptions of snoring as a potential indicator of underlying systemic health risks. The instrument is designed to identify critical gaps in public health literacy and to inform the development of targeted educational interventions. Ultimately, the scale facilitates a paradigm shift from reactive disease management to proactive, community-based prevention by leveraging snoring as an accessible entry point for early risk identification and health promotion.

## 2 Method

### 2.1 Phase 1 development of the scale

#### 2.1.1 Literature review

During the scale literature review, databases including CNKI, PubMed, Web of Science, Embase, and Scopus were searched using the following search terms: “snore,” “snoring,” “simple snoring,” “sleep apnea,” “obstructive sleep apnea,” “obstructive sleep apnea hypopnea syndrome,” “OSAHS,” “OSA,” “knowledge,” “attitude,” “behavior,” “knowledge-attitude-practice (KAP),” “questionnaire,” “tool,” and “scale.” Literature retrieval was conducted from database inception to October 28, 2022. After duplicate removal and screening of titles, abstracts, and full texts, 13 relevant studies were ultimately included. Subsequent synthesis of these included studies resulted in the formation of the source for the initial item pool.

#### 2.1.2 Cognitive interview

Purposive sampling was used to select community residents for semi-structured interviews (see Supplementary material 1 for the Interview Guide). The interviews aimed to explore their intuitive perceptions, daily attitudes, and related behavioral habits toward snoring (18).

In consideration of the different subtypes of snoring and the varying levels of awareness among community residents, we categorized the interview participants into three groups. Detailed inclusion and exclusion criteria are as follows:

① Non-snorers (19): individuals who report snoring almost never (≤1–2 times monthly); ② Snorers without medical consultation (19): Snorers without medical consultation: individuals who snore ≥1–2 times weekly but have not received a formal medical diagnosis or treatment, including those with primary snoring, habitual snoring, or suspected OSAHS with symptoms like gasping/choking during sleep; ③ Diagnosed OSAHS Patients (20): individuals with a confirmed OSAHS diagnosis (defined as ≥30 recurrent apneas/hypopneas per 7 h of sleep, or an apnea-hypopnea index [AHI] ≥ 5 events/h) who are currently in community care. All participants were required to meet the following criteria: provide informed consent to participate in the study; aged ≥18 years, able to independently comprehend and complete questionnaires, and voluntarily agree to participate in this research. Individuals with severe organ dysfunction or mental disorders were excluded.

Sample size was determined based on the principle of data saturation. A total of 30 participants were included, with 10 from each of three groups. After summarizing and analyzing the participants’ responses, the initial item pool was supplemented and revised further.

#### 2.1.3 Delphi consultation

Fifteen experts with experience in the field of sleep medicine, otolaryngology or psychology were invited to evaluate the content of the scale by paper questionnaire or email communication. The eligibility criteria for expert panelists were: (1) associate senior professional title or above with working experience of ≥10 years; (2) master’s degree or above; (3) extensive working experience in snoring-related medical care and scale development; and (4) participation in this study voluntarily. The assessment dimensions encompassed the content adequacy of the item pool and the precision of language expression, while a 5-point Likert scale was administered to evaluate the importance of individual items, and a designated blank section was reserved for experts to elaborate on their recommendations.

Questionnaires were retrieved within 14 days following their distribution, and members of the research team synthesized and analyzed experts’ feedback (21). The response rates for the two rounds of expert consultation were 93.75 and 100%, respectively; the individual experts’ authority coefficients ranged from 0.79 to 0.95, with an overall authority coefficient of 0.89, both of which exceeded the threshold of 0.70 (see Supplementary materials 2, 3 for Expert Information) (22). In the first round of expert consultation, five items with full-score ratios < 20% and coefficients of variation (CV) > 25% were eliminated based on expert feedback, six ambiguous or incompletely phrased items were revised, and three additional items were added to the knowledge dimension. In the second round of expert consultation, expert opinions showed a tendency toward consensus with good consistency, and no items were added, removed, or revised. Subsequently, the research team computed the scale-level content validity index (S-CVI) and item-level content validity index (I-CVI) based on experts’ responses to each item.

#### 2.1.4 Pilot study

A pilot test was performed in June 2023 to assess the item clarity and revise the scale in preparation for further surveys. Convenience sampling was adopted to select 30 adults for a pilot survey, with the same inclusion and exclusion criteria as those used for the semi-structured interviews. Feedback from participants toward the items was gathered, and based on the analysis of this feedback, two items underwent revision with respect to their linguistic expression.

### 2.2 Phase 2 evaluation of the scale’s psychometric properties

#### 2.2.1 Participants and setting

The formal survey targeted all adult residents in the community. Inclusion criteria were: (1) provide informed consent for the study; (2) aged ≥18 years; (3) possess the ability to independently comprehend and complete the questionnaires; and (4) voluntarily agree to participate in the research. Individuals with severe organ dysfunction or mental disorders were excluded.

The required sample size should be 10 to 20 times the total number of items (23). The number of items in this step was 49; therefore, the minimum sample size was estimated to be approximately 544, considering a 10.0% rate of invalid questionnaires.

#### 2.2.2 Data collection

A stratified sampling method was employed to select adults from urban and rural areas across four districts in Henan Province, China. The survey was conducted using both paper questionnaires completed on-site and online questionnaires through the platform Wenjuanxing between July, 2023, and September, 2023. This study was approved by the Ethics Committee of Zhengzhou University.

#### 2.2.3 Tools

The questionnaire was designed by the research team and included three sections (see Supplementary materials 4–7 for the Informed Consent Form for Participants and the Questionnaire): The first was a General Information Questionnaire, covering participants’ gender, age, training history, years of experience, professional title, and affiliated institution; the second section corresponds to the administration version of the Adult Snoring Knowledge, Attitude, and Practice (KAP) Scale. The Knowledge dimension encompasses 22 items, covering the etiology, causes and risk factors, detrimental effects, and knowledge on seeking medical care for adult snoring; the Attitude dimension consists of 11 items, focusing on attitudes toward monitoring nighttime sleep patterns and addressing adult snoring (including prevention and treatment); the Practice dimension includes 16 items, involving behaviors related to knowledge acquisition, self-health surveillance, and behaviors for preventing and mitigating adult snoring. Responses were scored using a 5-point Likert scoring method, with responses for the Knowledge dimension ranging from “Completely unaware” to “Very clear” (scored 1 to 5 accordingly), for the Attitude dimension from “Strongly disagree” to “Strongly agree” (also scored 1 to 5), and for the Practice dimension from “Strongly inconsistent” to “Strongly consistent” (scored 1 to 5); and the third was the Consumer Health Activation Index Scale, functioning as the criterion scale (24).

#### 2.2.4 Statistical methods

Data entry was performed using EpiData3.1, and data analysis was performed using SPSS27.0 and AMOS26.0. Item selection was conducted using the discriminant analysis, related analysis, homogeneity test, and internal consistency test. Then, samples were randomly divided into two subgroups based on the random numbers generated in STATA; one subgroup was utilized for exploratory factor analysis (EFA), and another was used for confirmatory factor analysis (CFA). Reliability of the scale was evaluated via internal consistency reliability, construct reliability and test–retest reliability; validity via content validity, convergent validity, discriminant validity and criterion validity.

## 3 Study results

### 3.1 Demographics

A total of 1,300 questionnaires were administered, yielding 1,198 valid responses, which resulted in a response rate of 92.1%. Sixty-one invalid questionnaires were excluded based on the criteria of selecting the same option and missing data, leaving 1,137 remaining valid questionnaires. In the total sample, one subgroup (n = 569) underwent item screening and exploratory factor analysis (EFA), while the other subgroup (*n* = 568) underwent confirmatory factor analysis (CFA), reliability, and validity testing. Demographic variables were comparable across the two subgroups, with no statistically significant differences observed. The basic characteristics of the participating population are shown in Table 1.

**Table 1:** Demographic characteristics of participants (*n* = 1,137).

Items		CFA *n* (%)		EFA *n* (%)		χ2	*p*
Gender	Female	275	(48.4)	282	(49.6)	0.15	0.699
	Male	293	(51.6)	287	(51.4)		
Age	18 ~ 29	239	(42.1)	209	(36.7)	3.43	0.180
	30 ~ 49	166	(29.2)	184	(32.3)		
	≥50	163	(28.7)	176	(31.0)		
Marital status	Unmarried	182	(32.1)	183	(32.2)	0.12	0.941
	Married	303	(53.3)	299	(52.5)		
	Others	83	(14.6)	87	(15.3)		
BMI	Normal	349	(61.4)	337	(59.2)	0.62	0.733
	Overweight	151	(26.6)	158	(27.8)		
	Obesity	68	(12.0)	74	(13.0)		
Education level	Primary and below	29	(5.1)	26	(4.6)	1.94	0.747
	Middle school	102	(18.0)	106	(18.6)		
	Junior college	146	(25.7)	161	(28.3)		
	Undergraduate	250	(44.0)	243	(42.7)		
	Master or above	41	(7.2)	33	(5.8)		
Percapita monthly household income	≤1,000	45	(7.9)	49	(8.6)	3.01	0.556
	1,001 ~ 2000	51	(9.0)	51	(9.0)		
	2001 ~ 3,000	110	(19.4)	108	(19.0)		
	3,001 ~ 4,000	111	(19.5)	132	(23.2)		
	>4,000	251	(44.2)	229	(40.2)		
Insurance payment type	Medical insurance for Urban Workers	267	(47.0)	291	(51.1)	1.98	0.372
	Commercial medical insurance for urban and rural residents	258	(45.4)	223	(39.2)		
	Insurance/self-paid	43	(7.6)	55	(9.7)		
Living arrangement	Alone	308	(54.2)	274	(48.2)	4.19	0.410
	With others	260	(45.8)	295	(51.8)		
Sleep quality	Very poor	95	(16.7)	91	(16.0)	1.18	0.882
	Poor	130	(22.9)	134	(23.5)		
	Average	168	(29.6)	157	(27.6)		
	Good	102	(18.0)	104	(18.3)		
	Very good	73	(12.8)	83	(14.6)		
Chronic disease	Yes	92	(16.2)	80	(14.1)	1.01	0.315
	No	476	(83.8)	489	(85.9)		
Snoring frequency	No snoring	217	(38.2)	198	(34.8)	0.54	0.910
	Occasionally (1–2 times/week)	192	(33.8)	197	(34.6)		
	Sometimes (3–5 times/week)	110	(19.4)	121	(21.3)		
	Frequently (6–7 times/week)	49	(8.6)	53	(9.3)		
Daytime Mental state	Very poor	46	(8.1)	40	(7.0)	2.32	0.676
	Poor	59	(10.4)	62	(10.9)		
	Average	237	(41.7)	259	(45.5)		
	Good	173	(30.5)	162	(28.5)		
	Very good	53	(9.3)	46	(8.1)		
Drinking frequency	Every day	17	(3.0)	28	(4.9)	5.052	0.282
	2–3 times a week	36	(6.3)	38	(6.7)		
	2–3 times a month	168	(29.6)	159	(27.9)		
	2–3 times a year	162	(28.5)	142	(25.0)		
	Almost never	185	(32.6)	202	(35.5)		
Smoking frequency	Every day	136	(24.0)	133	(23.4)	0.15	0.997
	2–3 times a week	33	(5.8)	35	(6.2)		
	2–3 times a month	32	(5.6)	31	(5.5)		
	2–3 times a year	29	(5.1)	28	(4.9)		
	Almost never	338	(59.5)	342	(60.1)		
Physical activity frequency	Never	82	(14.4)	71	(12.5)	2.40	0.663
	2–3 times a year	77	(13.6)	76	(13.3)		
	2–3 times a month	156	(27.5)	166	(29.2)		
	2–3 times a week	163	(28.7)	177	(31.1)		
	Every day	90	(15.8)	79	(13.9)		

### 3.2 Item analysis

#### 3.2.1 Discrimination analysis

All subjects were ranked in descending order of their total scores on the scale. The top 27.0% of subjects based on scores were assigned to the high-score group, while the bottom 27.0% were allocated to the low-score group (25). There were statistically significant differences in all items between the high-score group and the low-score group (*p* < 0.05), indicating strong discriminative power and that no items required elimination.

#### 3.2.2 Related analysis

The correlation coefficients between individual item scores and the total scale score ranged from 0.40 to 0.85, demonstrating each item was closely aligned with the core construct of the scale, with good discrimination and construct validity of the scale (26). In this study, items K7, K19, K20, A2, A3, A6, A11, P1, P2, P3, P5, P7, P9, P10, P11, and P16 were excluded as their correlation coefficients with the total scale score or their respective dimensions were < 0.40, see Table 2.

**Table 2:** Item analysis, reliability, and validity test of the 49 items.

Factor	Item	Discrimination index	Total correlation	Dimension correlation	CITC	Item deleted α	Decision	Cronbach^’^s α	CR	AVE	Standardized factor loading
F1	K1	1.89**	0.70**	0.66**	0.68	0.947	Retention	0.912	0.911	0.573	0.76
	K21	1.78**	0.68**	0.72**	0.66	0.951	Retention				0.76
	K22	1.68**	0.69**	0.74**	0.67	0.948	Retention				0.75
F2	K2	1.30**	0.63**	0.74**	0.61	0.948	Retention	0.917	0.917	0.553	0.72
	K3	1.26**	0.62**	0.74**	0.60	0.951	Retention				0.73
	K4	1.75**	0.68**	0.72**	0.66	0.948	Retention				0.74
	K5	1.30**	0.61**	0.71**	0.59	0.951	Retention				0.74
	K6	1.42**	0.63**	0.73**	0.60	0.948	Retention				0.70
	K8	1.90**	0.69**	0.68**	0.67	0.947	Retention				0.73
	K9	1.86**	0.69**	0.70**	0.67	0.952	Retention				0.72
	K10	1.66**	0.67**	0.72**	0.65	0.948	Retention				0.76
	K11	1.30**	0.61**	0.72**	0.59	0.948	Retention				0.76
F3	K12	1.39**	0.62**	0.72**	0.60	0.948	Retention	0.908	0.917	0.622	0.73
	K13	1.62**	0.69**	0.75**	0.67	0.947	Retention				0.80
	K14	1.56**	0.69**	0.76**	0.67	0.951	Retention				0.83
	K15	1.60**	0.67**	0.75**	0.65	0.948	Retention				0.79
	K16	1.56**	0.68**	0.76**	0.66	0.950	Retention				0.79
	K18	1.56**	0.69**	0.76**	0.67	0.949	Retention				0.79
F4	A1	2.23**	0.67**	0.79**	0.64	0.949	Retention	0.925	0.909	0.613	0.84
	A4	2.15**	0.54**	0.72**	0.51	0.949	Retention				0.64
	A5	2.28**	0.56**	0.74**	0.53	0.948	Retention				0.64
	A7	2.41**	0.70**	0.83**	0.67	0.949	Retention				0.88
	A8	2.42**	0.68**	0.82**	0.66	0.948	Retention				0.87
	A9	2.22**	0.66**	0.78**	0.64	0.949	Retention				0.81
	A10	2.25**	0.66**	0.79**	0.64	0.952	Retention				0.84
F5	P4	1.78**	0.63**	0.66**	0.60	0.949	Retention	0.923	0.910	0.634	0.74
	P6	2.16**	0.67**	0.70**	0.64	0.951	Retention				0.79
	P8	2.20**	0.67**	0.72**	0.65	0.948	Retention				0.80
	P12	2.17**	0.68**	0.73**	0.65	0.946	Retention				0.82
	P13	2.02**	0.65**	0.70**	0.63	0.952	Retention				0.79
	P14	2.13**	0.70**	0.74**	0.68	0.949	Retention				0.85
	P15	2.30**	0.65**	0.72**	0.63	0.951	Retention				0.84
	K7	0.76**	0.33**	0.42**	0.30	0.948	Deletion				
	K17	0.47**	0.27**	0.48**	0.23	0.949	Deletion				
	K19	0.40**	0.23**	0.40**	0.19	0.952	Deletion				
	K20	0.33**	0.20**	0.39**	0.17	0.949	Deletion				
	A2	0.74**	0.32**	0.37**	0.29	0.951	Deletion				
	A3	0.82**	0.32**	0.43**	0.30	0.948	Deletion				
	A6	0.83**	0.31**	0.41**	0.28	0.949	Deletion				
	A11	0.86**	0.35**	0.46**	0.26	0.952	Deletion				
	P1	0.79**	0.30**	0.36**	0.26	0.949	Deletion				
	P2	0.70**	0.30**	0.38**	0.27	0.951	Deletion				
	P3	0.41**	0.20**	0.28**	0.17	0.948	Deletion				
	P5	0.73**	0.29**	0.38**	0.26	0.949	Deletion				
	P7	1.16**	0.39**	0.49**	0.31	0.952	Deletion				
	P9	1.22**	0.32**	0.44**	0.28	0.948	Deletion				
	P10	1.29**	0.34**	0.46**	0.30	0.951	Deletion				
	P11	0.82**	0.32**	0.43**	0.29	0.949	Deletion				
	P16	0.77**	0.34**	0.43**	0.31	0.949	Deletion				

CR, Critical ratio; **p < 0.001; CV, coefficient of variation; CITC, Corrected Item-Total Correlation.

#### 3.2.3 Homogeneity test

No notable increase in Cronbach^’^s *α* was observed after removing any item. However, items K7, K17, K20, A2, A3, A6, A11, P1, P2, P3, P5, P7, P9, P10, P11, and P16 had a corrected item-total correlation < 0.40 and were therefore excluded (25), as shown in Table 2.

### 3.3 Exploratory factor analysis

The KMO value of this study was 0.90, the Bartlett spherical test was statistically significant (χ^2^ = 7810.005, *p* < 0.001), and the data were suitable for exploratory factor analysis (25). Principal component analysis (PCA) was used to extract common factors, followed by varimax rotation for orthogonal transformation (25). Five common factors with eigenvalues > 1 were extracted, accounting for a cumulative variance explained of 64.038%. After rotation, all factor loadings exceeded 0.40, with each factor containing more than three items (27). Following discussion, the scale ultimately retained five factors, as shown in Table 3.

**Table 3:** Exploratory factor analysis of the total scale.

Item number	Items	1	2	3	4	5
K1	Frequent snoring may be a disease state					0.60
K2	Hypothyroidism can cause snoring				0.67	
K3	Chronic heart failure can cause snoring				0.70	
K4	Lesions in the nose and throat can cause snoring				0.55	
K5	People with a receding chin (commonly known as “beak,” where most of the gums are exposed when smiling, and the teeth are exposed when the mouth is slightly opened) tend to snore				0.69	
K6	People with shorter necks are more likely to snore				0.74	
K8	Middle-aged and older adults are more likely to snore				0.42	
K9	Heavy alcohol consumption can lead to snoring				0.47	
K10	Long-term smoking can aggravate snoring				0.57	
K11	Long-term use of sedatives, hypnotics, or muscle relaxants (drugs that relax muscles) can lead to snoring				0.69	
K12	Snoring can damage the digestive systems (such as causing xerostomia, bitter taste in the mouth, acid regurgitation and heartburn, etc.)	0.75				
K13	Snoring can damage the cardiovascular system (such as causing high blood pressure, pulmonary heart disease, coronary heart disease and irregular heartbeats, etc.)	0.77				
K14	Snoring harms the nervous system (such as causing stroke, mania, and depression, etc.)	0.85				
K15	Snoring can damage the respiratory system (such as aggravating chronic bronchitis, etc.)	0.81				
K16	Snoring can lead to metabolic disorders (such as hyperlipidemia and diabetes, etc.)	0.87				
K18	Snoring can easily lead to a decline in memory	0.79				
K21	Simple snoring may progress to a more severe stage without intervention					0.64
K22	The diagnosis of snoring requires sleep monitoring at the hospital					0.70
A1	Pay attention to snoring during sleep		0.62			
A4	Quitting smoking is beneficial for improving snoring		0.79			
A5	Quitting alcohol is beneficial for improving snoring		0.80			
A7	Effective treatment of heart failure is beneficial for improving snoring		0.87			
A8	Timely treatment of nasal and throat diseases is beneficial for improving snoring		0.84			
A9	Seek medical attention if you notice yourself snoring		0.74			
A10	Encourage family members or friends who snore to seek medical attention		0.67			
P4	I often check with family about changes in my snoring while sleeping			0.52		
P6	I pay attention to my memory condition			0.44		
P8	If I have nasopharyngeal and laryngeal diseases, I will seek timely medical attention			0.58		
P12	If I develop snoring or my snoring worsens, I will seek medical attention			0.80		
P13	If I experience daytime fatigue and drowsiness, I will seek medical attention			0.76		
P14	If I frequently experience a dry mouth and bitter taste in the morning, I will seek medical attention			0.81		
P15	If I frequently experience acid reflux and heartburn, I will seek medical attention			0.77		

### 3.4 Confirmatory factor analysis

**Figure 1:**
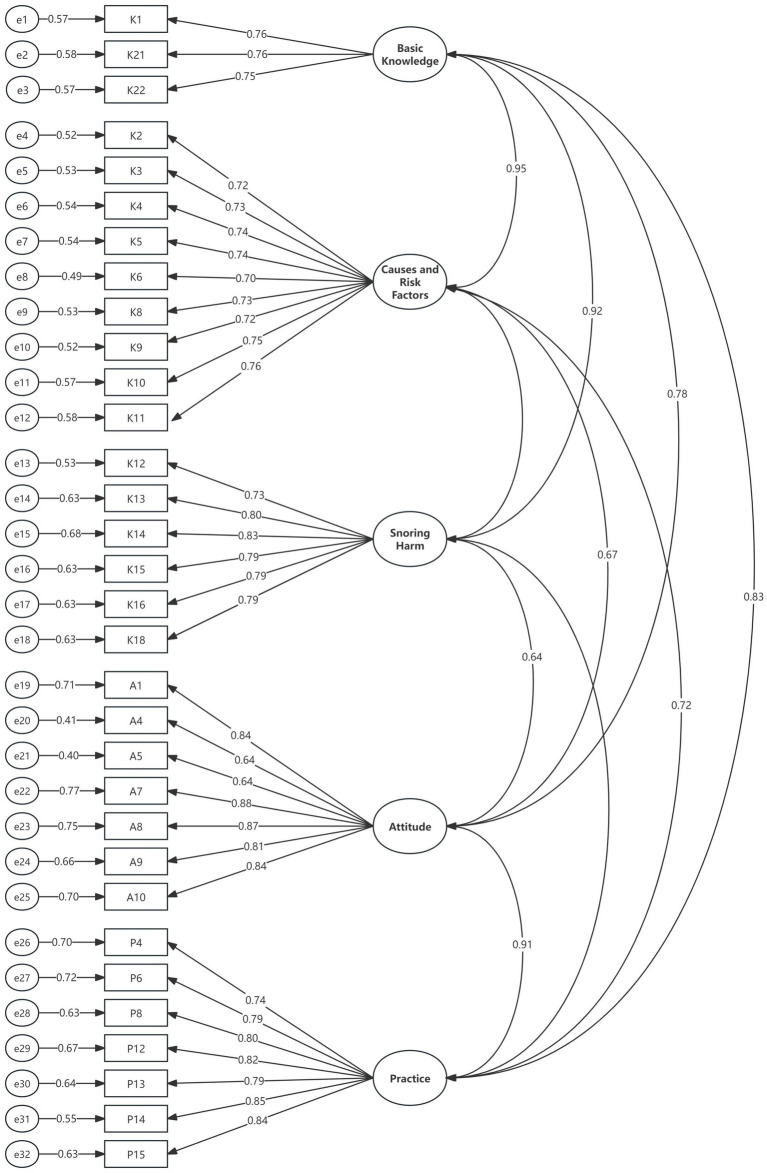
Confirmatory factor analysis model of community residents’ knowledge-attitude-practice toward adult snoring.

Based on the Knowledge, Attitude, and Practice (KAP) theory, confirmatory factor analysis (CFA) was conducted on the five-factor model. All fit indices of the model were found to meet the established criteria, exhibiting good fit. This confirms that the factor structure derived from exploratory factor analysis (EFA) and its relationship with the corresponding measurement items exist and are highly stable. The model fit indices and criteria are presented in Table 4, and the factor model is illustrated in Figure 1.

**Table 4:** Model fit indices and reliability of community residents’ knowledge-attitude-practice toward adult snoring.

Dimension	Items number	χ^2^/df	RMSEA	CFI	GFI
Knowledge	18	2.740	0.050	0.953	0.920
Attitude	7	2.907	0.050	0.974	0.961
Practice	7	2.240	0.041	0.985	0.973
Total	32	2.765	0.059	0.925	0.910
Standard value		<3.000	≤0.080	>0.900	>0.900

CFI, comparative fit index; χ^2^/df, Chi-square value/degrees of freedom; GFI, goodness of fit index; RMSEA, root-mean-square error of approximation.

### 3.5 Reliability

#### 3.5.1 Internal consistency reliability

Cronbach’s *α* was employed to assess the internal consistency of the scale, reflecting its internal reliability. The Cronbach’s α for the total scale was 0.96, with the knowledge, attitude, and practice subscales demonstrating coefficients of 0.95, 0.93, and 0.92, respectively; all values exceeded 0.90 (28).

#### 3.5.2 Construct reliability

The construct reliability (CR) values of the five factors were 0.911, 0.912, 0.908, 0.920, and 0.910, respectively, all meeting the criterion of > 0.8, indicating good construct reliability of the scale (29).

#### 3.5.3 Test–retest

Eighty-eight participants were selected for retesting 2 weeks later, with a test–retest reliability of 0.88 exceeding the criterion of 0.7, indicating that the scale had high test–retest reliability and good stability (30).

### 3.6 Validity

#### 3.6.1 Content validity

Calculated based on expert ratings of the importance of each scale item and its relevance to the corresponding content (1–4, 1 = very irrelevant and 4 = very relevant), the scale’s content validity (S-CVI) was 0.93, meeting the criterion of >0.90. The item-level content validity (I-CVI) ranged from 0.81 to 0.93, all exceeding the criterion of 0.78 (31).

#### 3.6.2 Convergent validity

Factor analysis was used to assess convergent validity, by examining the loadings of each item on its respective factor and calculating the ratio of the sum of variances of items under each factor to the factor’s total variance. For this scale, item loadings on their respective factors ranged from 0.64 to 0.88 (all > 0.50), with the average variance extracted (AVE) for all latent variables >0.50 and construct reliability (CR) values for both subscales and the total scale >0.80, indicating good convergent validity (32), see Table 2.

#### 3.6.3 Discriminant validity

Compare the square root of each factor’s average variance extracted (AVE) with its correlation coefficients with other factors. If the square root of a factor’s AVE is greater than its correlation with any other factor, it indicates good discriminant validity for that factor. In this study, all factors showed significant correlations, and the square root of each factor’s AVE was greater than its correlations with other factors (30), as shown in Table 5.

**Table 5:** The discriminant validity of each factor.

Factor	F1	F 2	F3	F4	F5
F1	1.00				
F2	0.70^**^	1.00			
F3	0.75^**^	0.72^**^	1.00		
F4	0.62^**^	0.56^**^	0.53^**^	1.00	
F5	0.68^**^	0.62^**^	0.57^**^	0.73^**^	1.00
The square root of AVE	0.76	0.74	0.79	0.78	0.79

^**^p < 0.001; AVE: the average variance extracted for each dimension; F1: basic cognition; F2: causes and risk factors; F3: snoring harm; F4: attitude; F5: practices.

#### 3.6.4 Criterion validity

The Consumer Health Activation Index Scale was used as the criterion to assess the criterion-related validity of this study’s scale. This criterion scale has a universal target population, comprises three dimensions (Knowledge, Self-Efficacy, and Action), and demonstrates good overall reliability and validity. The correlation coefficient between this study’s scale and the criterion scale was 0.41 (*p* < 0.01), which falls within the ideal range of 0.4 ~ 0.8 for criterion-related validity (26).

### 3.7 Critical values

The total scale consisted of 32 items (Supplementary material 8), all scored on a 5-point Likert scale. The total scale score was calculated by summing the total scores of each subscale, with a score range of 32–160 points. Based on the actual scores of the study subjects, K-means cluster analysis was used to classify the total scale scores, which were ultimately divided into three levels: low (32–86 points), moderate (87–109 points), and high (110–160 points).

## 4 Discussion

To our knowledge, this is the first validated instrument to reframe snoring as a community-perceivable indicator of systemic health risk. By grounding assessment in daily experience, this scale fills a critical void in public health tools: it enables early engagement across the entire community adult population. The robust psychometric performance of the scale further supports its utility in identifying knowledge gaps, attitudinal barriers, and actionable opportunities for health promotion in real world settings.

Existing instruments for sleep-disordered breathing, such as the Berlin Questionnaire and STOP-Bang (33, 34), were developed primarily for clinical screening and risk stratification of OSA in symptomatic individuals. In contrast, this scale deliberately targets the preclinical stage of risk awareness in the general population, shifting the focus from the severe end of the snoring-OSAHS spectrum to earlier stages and non-snoring residents. It aims to enhance risk awareness and promote proactive behaviors among the general population, as well as primary prevention and family health supervision for non-snoring residents at the community level (35).

This scale can generate actionable insights for community-based interventions. For instance, a subcategory with high knowledge but low practice scores may indicate structural barriers such as limited access to weight management programs or sleep health education. Importantly, the scale also captures the role of family members as facilitators of early action, enabling interventions extending beyond the individual to the household unit. Such features make the instrument suited for integration into community health worker programs, or digital health platforms targeting modifiable risk behaviors before chronic conditions manifest.

### 4.1 Strengths and limitations

This study regards adult snoring as a commonly perceived phenomenon systemic health risk. This community perspective enables community-level assessment risk perceptions and behavioral tendencies about snoring before health issues become prominent, thus supporting timely public health promotion initiatives. Furthermore, its modular design allows flexible use: it can be administered as a complete instrument or with subscale scores for knowledge, attitudes, and practices separately to address specific assessment.

This study was conducted based on the KAP theoretical framework, and no empirical tests were carried out on the causal relationships among its various dimensions. In addition, the instrument was developed in Chinese context, future research across diverse countries and regions is needed to verify these findings and explore broader applicability.

## 5 Conclusion

This study employed a rigorous scale adaptation process that included qualitative data, expert collaboration, and statistical analysis. The multifaceted methodological approach guarantees the scale’s scientific rigor, clinical utility, and good reliability and validity. This scale enables healthcare professionals and public health professionals to conduct quantitative evaluation toward Adult Snoring in community settings. The findings can inform tailored health education initiatives and behavior modification interventions to promote early risk awareness and preventive action.

## Data Availability

The original contributions presented in the study are included in the article/Supplementary material, further inquiries can be directed to the corresponding author/s.
