# Supplementary material for: Development and validation of a scale for assessing community adults’ knowledge, attitude and practice toward adult snoring
Source: Front Public Health. 2026 Mar 10;14:1754193. doi: 10.3389/fpubh.2026.1754193 (PMC13014041; doi:10.3389/fpubh.2026.1754193)
Supplement: Supplementary file 1 [file Data_Sheet_1.PDF]

Supplementary Material 1

Interview Guide

Participants and Inclusion/Exclusion Criteria

This interview involved three groups of participants: (1) Non-snorers; (2) Snorers without medical consultation; (3) Diagnosed OSAHS Patients.

Inclusion Criteria for the Three Groups:

①Non-snorers: individuals who report snoring almost never (≤1-2 times monthly); ②Snorers without medical consultation: individuals who snore ≥1-2 times weekly but have not received a formal medical diagnosis or treatment (including those with primary snoring, habitual snoring, or suspected OSAHS with symptoms like gasping/choking during sleep; ③Diagnosed OSAHS Patients: individuals with a confirmed OSAHS diagnosis (defined as ≥30 recurrent apneas/hypopneas per 7 hours of sleep, or an apnea-hypopnea index [AHI] ≥5 events/hour) who are currently in community care.

All participants were required to meet the following criteria: provide informed consent to participate in the study; aged ≥18 years, able to independently comprehend and complete questionnaires, and voluntarily agree to participate in this research. Individuals with severe organ dysfunction or mental disorders were excluded.

①Do you usually snore? If yes, what is the frequency of your snoring?

②What do you know about snoring? Through what channels did you learn about "snoring"?

③What health impacts do you think snoring may have?

④How much do you know about the causes and common symptoms of snoring?

⑤Do you think adult snoring is a health issue that needs attention?

⑥Do you take the initiative to learn health-related knowledge? After learning, do you adjust your health behaviors?

⑦Do you think it is necessary to popularize snoring knowledge among ordinary residents? If necessary, what content should be the focus of popularization?

⑧How do you evaluate your overall health status? Do you take the initiative to learn health-related knowledge and adjust your behaviors accordingly?

⑨What methods do you think can be used in daily life to prevent snoring or avoid its

aggravation?
⑩Besides the above topics, do you have any additional views or suggestions on other aspects related to snoring?
⑪What do you think of measures such as adjusting lifestyle and medical auxiliary methods to improve or prevent snoring? Are you willing to try them?
⑫If you snore, how long has this situation lasted? Have you ever experienced discomfort such as nighttime awakening due to suffocation, morning headache, or daytime sleepiness?
⑬If you snore or have related discomfort, have you ever received professional diagnosis in the hospital? What are the reasons for not being diagnosed / having been diagnosed?
⑭Do you think adult snoring requires medical intervention? Should different treatment methods be adopted for snoring of different severity?

Supplementary Material 2

Table 1 Basic Information of consulted experts, (*n*=15)

Number	Age	Education	Professional Title	Specialty Area	Working years
1	53	Master degree	Chief physician	Respiratory-sleep department	30
2	50	Master degree	Associate chief nurse	Otolaryngology-Head and Neck Surgery	31
3	42	Doctoral degree	Associate chief physician	Otolaryngology-Head and Neck Surgery	16
4	43	Master degree	Associate chief nurse	Respiratory-sleep department	16
5	45	Doctoral degree	Associate chief physician	Otolaryngology-Head and Neck Surgery	21
6	46	Doctoral degree	Associate chief physician	Respiratory-sleep department	19
7	43	Doctoral degree	Chief physician	Otolaryngology-Head and Neck Surgery	17
8	49	Doctoral degree	Associate chief physician	Otolaryngology-Head and Neck Surgery	25
9	40	Doctoral degree	Chief physician	Respiratory-sleep department	15
10	46	Doctoral degree	Associate chief physician	Respiratory-sleep department	19
11	49	Doctoral degree	Chief physician	Otolaryngology-Head and Neck Surgery	25
12	54	Master degree	Associate chief nurse	Respiratory-sleep department	35
13	46	Doctoral degree	Chief physician	Otolaryngology-Head and Neck Surgery	26
14	40	Doctoral degree	Chief nurse	Respiratory-sleep department	15
15	50	Doctoral degree	Professor	Mental health	30

Supplementary Material 3

Table 2 Authority coefficient of consulted experts, (*n*=15)

Number	q1	q2	q3	Total	Expert authority coefficient
1	1.00	0.90	0.73	2.63	0.88
2	0.80	0.90	0.80	2.50	0.83
3	0.80	0.80	0.80	2.40	0.80
4	0.80	0.85	0.80	2.45	0.82
5	0.80	0.90	0.68	2.38	0.79
6	0.80	0.90	1.00	2.70	0.90
7	1.00	0.90	0.72	2.62	0.87
8	0.80	0.85	0.80	2.45	0.82
9	1.00	0.85	0.90	2.75	0.92
10	0.80	0.80	0.92	2.52	0.84
11	1.00	0.85	0.80	2.65	0.88
12	0.80	0.85	0.96	2.61	0.87
13	1.00	0.85	1.00	2.85	0.95
14	1.00	0.90	0.90	2.8	0.93
15	1.00	0.95	0.60	2.55	0.85

Supplementary Material 4

Informed Consent Form for Participants

Dear Participants,

We are researchers from the School of Nursing and Health, Zhengzhou University, conducting a study titled "Development and Application of a Knowledge, Attitude, and Practice Scale for Adult Snoring". The aim of this research is to develop a scientific assessment tool for adult snoring-related knowledge, attitudes, and behaviors. We sincerely invite you to participate by completing a short questionnaire-your perspectives and experiences will greatly contribute to our study.

Key Details for Your Reference:① The questionnaire takes approximately 10 minutes to complete;② All your responses are used solely for academic research purposes. Your personal information will be fully anonymized and strictly confidential, with no disclosure to third parties;③ Participation is entirely voluntary. Your involvement will not have any adverse impact on you, and you may withdraw from the survey at any time without providing a reason or facing any negative consequences.

If you agree to participate in this study, please sign below to confirm your informed consent. Thank you for your valuable support!

I have read and fully understood the above information, including the study purpose, participation requirements, and my rights. I confirm that my participation is voluntary, with no adverse impact, and I have the right to withdraw at any time.

□ I agree to participate in this study

□ I decline to participate

Signature: ________________________

Date:

Supplementary Material 5

General Information Questionnaire

Dear Participants,

Hello! This survey aims to understand your knowledge and attitudes towards adult snoring. Your honest responses are crucial to our research. Please follow the below instructions to complete the questionnaire. Wish you a pleasant life!

Instructions for Completion:

This survey is anonymous—your personal information and responses will not be disclosed to any third party.

There are no right or wrong answers. Please answer based on your actual situation.

For each question, select only one option and mark "√" in the corresponding box. Do not skip items or leave blanks.

If you need to fill in text (marked "________"), please write clearly and concisely.

Estimated completion time: 10 minutes. Thank you for your cooperation!

1. Gender: ① Male ② Female

2. Age: ① 18-29 ② 30-49 ③ ≥50

3. Marital Status: ① Unmarried ② Married ③ Others

4. Height & Weight (for BMI calculation):

Height: ________ cm   Weight: ________ kg

5. Education level: ① primary and below ② middle school ③ junior college ④ undergraduate ⑤ master or above

6. Per capita monthly household income: ① ≤1000 yuan ② 1001~2000 yuan ③ 2001~3000 yuan ④ 3001~4000 yuan ⑤ ＞4000 yuan

7. Insurance payment type: ① medical insurance for Urban Workers ② commercial medical insurance for urban and rural residents ③ insurance/self-paid

8. Living Arrangement: ① Alone ② With Others

9. Sleep Quality: ① Very Poor ② Poor ③ Average ④ Good ⑤ Very Good

10. Chronic Disease : ① Yes ② No

11. Snoring Frequency: ① No snoring ② Occasionally (1-2 times/week) ③

Sometimes (3-5 times/week) ④ Frequently (6-7 times/week)

12. Daytime Mental State: ① Very Poor ② Poor ③ Average ④ Good ⑤ Very Good

13. Drinking frequency: ① Daily ② 2-3 times a week ③ 2-3 times a month ④ 2-3 times a year ⑤ Almost Never

14. Smoking Frequency: ① Dvery day ② 2 to 3 times a week ③ 2 to3 times a month ④ 2 to 3 times a year ⑤ Almost Never

15. Physical Activity Frequency: ① Never ② 2 to 3 times a year ③ 2 to 3 times a month ④ 2 to 3 times a week ⑤ Every day

Supplementary Material 6

Table 3 Adult Snoring Knowledge, Attitude, and Practice (KAP) Scale (Administered version)

Knowledge Dimension

Responses were scored using a 5-point Likert scale, ranging from "Completely unaware" to "Very clear" (scored 1 to 5 accordingly).

Item		1	2	3	4	5
K1	Frequent snoring may be a disease state					
K2	Hypothyroidism can cause snoring					
K3	Chronic heart failure can cause snoring					
K4	Lesions in the nose and throat can cause snoring					
K5	People with a receding chin (commonly known as" beak ," where most of the gums are exposed when smiling, and the teeth are exposed when the mouth is slightly opened) tend to snore					
K6	People with shorter necks are more likely to snore					
K7	Obese individuals are at an increased risk of snoring.					
K8	Middle-aged and older adults are more likely to snore					
K9	Heavy alcohol consumption can lead to snoring					
K10	Long-term smoking can aggravate snoring					
K11	Long-term use of sedatives, hypnotics, or muscle relaxants (drugs that relax muscles) can lead to snoring					
K12	Snoring can damage the digestive systems (such as causing xerostomia, bitter taste in the mouth, acid regurgitation and heartburn, etc.)					
K13	Snoring can damage the cardiovascular system (such as causing high blood pressure, pulmonary heart disease,coronary heart disease and irregular heartbeats, etc.)					
K14	Snoring harms the nervous system (such as causing stroke, mania, and depression, etc.)					
K15	Snoring can damage the respiratory system (such as aggravating chronic bronchitis, etc.)					
K16	Snoring can lead to metabolic disorders (such as hyperlipemia and diabetes, etc.)					
K17	Snoring can easily lead to daytime fatigue and drowsiness, affecting daytime work and life					
K18	Snoring can easily lead to a decline in memory					
K19	Snoring requires medical consultation with the Department of Respiratory and Sleep Medicine or the Department of Otolaryngology-Head and Neck					

	Surgery in a hospital.					
K20	Chang sleeping position (such as from lying on the back to on the side) can improve snoring					
K21	Simple snoring may progress to a more severe stage without intervention					
K22	The diagnosis of snoring requires sleep monitoring at the hospital					

Attitude Dimension

Responses were scored using a 5-point Likert scale, ranging from "Strongly disagree" to "Strongly agree" (scored 1 to 5 accordingly).

**Item**		**1**	**2**	**3**	**4**	**5**
A1	Pay attention to snoring during sleep					
A2	Pay attention to whether the energy recovery after waking up in the morning					
A3	Body weight needs to be controlled in the normal range					
A4	Quitting smoking is beneficial for improving snoring					
A5	Quitting alcohol is beneficial for improving snoring					
A6	Treating brain and other nervous system disorders (such as stroke) is beneficial for improving snoring					
A7	Effective treatment of heart failure is beneficial for improving snoring					
A8	Timely treatment of nasal and throat diseases is beneficial for improving snoring					
A9	Seek medical attention if you notice yourself snoring					
A10	Encourage family members or friends who snore to seek medical attention					
A11	It is hoped that relevant organizations and media will popularize knowledge about snoring among community residents					

Practice Dimension

Responses were scored using a 5-point Likert scale, ranging from "Strongly inconsistent" to "Strongly consistent" (scored 1 to 5 accordingly).

**Item**		**1**	**2**	**3**	**4**	**5**
P1	I actively attend community health promotion campaigns to learn about snoring					
P2	I pay attention my sleep at night					
P3	I regularly inquire of my family whether I snore during sleep					
P4	I often check with family about changes in my snoring while sleeping					
P5	I pay attention to whether I feel excessively sleepy during the day					
P6	I pay attention to my memory condition					
P7	I exercise regularly and focus on weight management					
P8	If I have nasopharyngeal and laryngeal diseases, I will seek timely					

	medical attention
P9	I don't smoke or plan to quit smoking
P10	I don't drink or plan to quit drinking
P11	If I have heart disease, I will seek timely medical treatment
P12	If I develop snoring or my snoring worsens, I will seek medical attention
P13	If I experience daytime fatigue and drowsiness, I will seek medical attention
P14	If I frequently experience a dry mouth and bitter taste in the morning, I will seek medical attention
P15	If I frequently experience acid reflux and heartburn, I will seek medical attention
P16	If medical staff recommend treatment for my snoring, I will accept the treatment advice

Supplementary Material 7

Please indicate how much you agree with each statement using the scale below.

Table 4 The Consumer Health Activation Index Scale

Item		1	2	3	4	5
1	I know how to find reliable health information when I need it.					
2	I feel confident talking to healthcare providers about my health concerns.					
3	I take steps to prevent health problems before they occur.					
4	I follow medical advice even when I don't feel well.					
5	I ask questions to make sure I understand my treatment options.					
6	I track my health status (e.g., blood pressure, weight) regularly.					
7	I work with healthcare providers to set health goals.					
8	I advocate for myself when navigating the healthcare system.					
9	I make healthy lifestyle choices (e.g., diet, exercise) to manage my health.					
10	I feel in control of my health and healthcare decisions.					

Supplementary Material 8

The final version of the Adult Snoring Knowledge, Attitude, and Practice (KAP) Scale consists of 32 items across three dimensions. The Knowledge dimension includes 18 items, covering basic cognition, risk factors, and disease-related harms of adult snoring; the Attitude dimension consists of 7 items; and the Practice dimension includes 7 items. Responses were scored using a 5-point Likert scale, with responses for the Knowledge dimension ranging from "Completely unaware" to “Very clear” (scored 1 to 5 respectively), for the Attitude dimension from “Strongly disagree” to “Strongly agree” (scored 1 to 5), and for the Practice dimension from "Strongly inconsistent" to “Strongly consistent” (scored 1 to 5). The total score of this scale ranges from 32 to 160, with the low-level score range of 32 to 86, the moderate-level score range of 87 to 109, and the high-level score range of 110 to 160.

In the knowledge dimension:K1, K21, and K22 correspond to Basic cognition; K2, K3, K4, K5, K6, K8, K9, K10and K11 correspond to Risk factor; K12, K13, K14, K15, K16 and K18 correspond to Snoring harm.

Table 5 Adult Snoring Knowledge, Attitude, and Practice (KAP) Scale (Final Version)

Items	Items in English	Items in Chinese
K1	Frequent snoring may be a disease state	经常打鼾可能是一种疾病状态
K2	Hypothyroidism can cause snoring	甲状腺功能减退会导致打鼾
K3	Chronic heart failure can cause snoring	慢性心力衰竭会导致打鼾
K4	Lesions in the nose and throat can cause snoring	鼻咽喉部位的病变会导致打鼾
K5	People with a receding chin (commonly known as "beak," where most of the gums are exposed when smiling, and the teeth are exposed when the mouth is slightly opened) tend to snore	下巴后缩者（俗称“鸟嘴”，微笑时会露出大部分牙龈，微微张嘴即可露出牙齿）容易打鼾
K6	People with shorter necks are more likely to snore	颈部较短的人容易打鼾
K8	Middle-aged and older adults are more likely to snore	中老年人，年龄越大越容易打鼾
K9	Heavy alcohol consumption can lead to snoring	大量饮酒易导致打鼾
K10	Long-term smoking can aggravate snoring	长期吸烟可加重打鼾
K11	Long-term use of sedatives, hypnotics, or muscle relaxants (drugs that relax muscles) can lead to snoring	长期服用镇静、催眠类或肌肉松弛类药物（使肌肉松弛的药物）会导致打鼾
K12	Snoring can damage the digestive systems (such as causing xerostomia, bitter	鼾症危害消化系统(如导致口干、口苦、反酸、烧

	taste in the mouth, acid regurgitation and heartburn, etc.)	心)
K13	Snoring can damage the cardiovascular system (such as causing high blood pressure, pulmonary heart disease, coronary heart disease and irregular heartbeats, etc.)	鼾症危害心血管系统(如导致高血压、肺源性心脏病、冠心病、心律失常等)
K14	Snoring harms the nervous system (such as causing stroke, mania, and depression, etc.)	鼾症危害神经系统(如导致中风、狂躁、抑郁等)
K15	Snoring can damage the respiratory system (such as aggravating chronic bronchitis, etc.)	鼾症危害呼吸系统(如可加重慢性支气管炎等)
K16	Snoring can lead to metabolic disorders (such as hyperlipemia and diabetes, etc.)	鼾症导致代谢紊乱(如高血脂、糖尿病等)
K18	Snoring can easily lead to a decline in memory	鼾症易造成记忆力下降
K21	Simple snoring may progress to a more severe stage without intervention	单纯的打鼾如果不进行干预治疗，可能会逐渐发展到更严重的阶段
K22	The diagnosis of snoring requires sleep monitoring at the hospital	鼾症的确诊需到医院做睡眠监测
A1	Pay attention to snoring during sleep	需要关注夜间的打鼾情况
A4	Quitting smoking is beneficial for improving snoring	戒烟对改善打鼾有益
A5	Quitting alcohol is beneficial for improving snoring	戒酒对改善打鼾有益
A7	Effective treatment of heart failure is beneficial for improving snoring	有效治疗心力衰竭对改善打鼾有益
A8	Timely treatment of nasal and throat diseases is beneficial for improving snoring	及时治疗鼻咽喉部疾病对改善打鼾有益
A9	Seek medical attention if you notice yourself snoring	发现自己打呼噜时及时去医院就诊
A10	Encourage family members or friends who snore to seek medical attention	发现家人或朋友打呼噜时，建议其去医院就诊
P4	I often check with family about changes in my snoring while sleeping	我经常询问家人自己睡眠时鼾声是否有所变化
P6	I pay attention to my memory condition	我关注自己的记忆力情况
P8	If I have nasopharyngeal and laryngeal diseases, I will seek timely medical attention	如果我出现鼻咽喉部疾病时，会及时看医生
P12	If I develop snoring or my snoring worsens, I will seek medical attention	如果我出现打鼾或打鼾加重时，我会去看医生
P13	If I experience daytime fatigue and drowsiness, I will seek medical attention	如果我出现白天疲乏嗜睡时，我会去看医生
P14	If I frequently experience a dry mouth and bitter taste in the morning, I will seek medical attention	如果我晨起经常口干、口苦，我会去看医生
P15	If I frequently experience acid reflux and heartburn, I will seek medical attention	如果我经常反酸烧心，我会去看医生
